# Selective Inhibition of Bakuchicin Isolated from* Psoralea corylifolia* on CYP1A in Human Liver Microsomes

**DOI:** 10.1155/2016/5198743

**Published:** 2016-02-09

**Authors:** Sun Joo Kim, Heung Chan Oh, Youn-Chul Kim, Gil-Saeng Jeong, Sangkyu Lee

**Affiliations:** ^1^BK21 Plus KNU Multi-Omics based Creative Drug Research Team, College of Pharmacy and Research Institute of Pharmaceutical Sciences, Kyungpook National University, Daegu 41566, Republic of Korea; ^2^College of Pharmacy, Wonkwang University, Iksan 54538, Republic of Korea; ^3^College of Pharmacy, Keimyung University, Daegu 42601, Republic of Korea

## Abstract

Bakuchicin is a furanocoumarin isolated from* Psoralea corylifolia* and shows several biological activities. Although there have been studies on the biological effects of bakuchicin, its modulation potency of CYP activities has not been previously investigated. Here, we investigated the inhibitory effects of bakuchicin on the activities of CYP isoforms by using a cocktail of probe substrates in pooled human liver microsomes (HLMs) and human recombinant* cDNA-expressed* CYP. Bakuchicin strongly inhibited CYP1A-mediated phenacetin* O*-deethylation with an IC_50_ value of 0.43 *μ*M in HLMs. It was confirmed by human recombinant* cDNA-expressed* CYP1A1 and CYP1A2 with a *K*_*i*_ value of 0.11 *μ*M and 0.32 *μ*M, respectively. A Lineweaver-Burk plot indicated that the inhibition mechanism of bakuchicin was competitive inhibition. Overall, this is the first study to investigate the potential CYP1A1 and CYP1A2 inhibition associated with bakuchicin and to report its competitive inhibitory effects on HLMs.

## 1. Introduction

Cytochrome P450 (CYP) is a group of heme-containing enzymes embedded essentially in the lipid bilayer of the endoplasmic reticulum, and it acts in the metabolism of many drugs, steroids, and carcinogens [1]. They mainly contribute to the biotransformation of xenobiotics from a hydrophobic state to a more readily extractable hydrophilic form [2]. The regulations of CYP activities are the major cause of drug-drug or herb-drug interactions [3]. Moreover, the control of drug-metabolizing enzymes frequently causes adverse drug effects during cancer therapy [4].

The CYP1A subfamily consisted of two members CYP1A1 and CYP1A2. CYP1A is one of the important CYP subfamilies in humans and plays a role in the metabolism of some clinically used drugs including theophylline, clozapine, tacrine, and foodborne procarcinogens [5]. The CYP1A subfamily consisted of two members CYP1A1 and CYP1A2. CYP1A1 is found mainly in extra hepatic tissues such as lung, gastrointestinal tract, placenta, and skin [6]. CYP1A1 is not significantly expressed in the liver, whereas CYP1A2 is expressed mainly in the liver [7]. However, polycyclic aromatic hydrocarbons (PAHs) significantly induce the expression of CYP1A1 in the liver, lung, kidney, and gut of rats [8]. CYP1A1, a well-known aryl hydrocarbon hydroxylase, is involved in the metabolic activation of environmental procarcinogens. CYP1A2, one of the major CYPs in the human liver (~13%), responds to the metabolism of a variety of arylamines and heterocyclic arylamines in therapeutic drugs [9, 10]. In particular, the CYP1A subfamily has historically been thought to play important roles in the bioactivation of many procarcinogens including PAHs, heterocyclic aromatic amines, and mycotoxins [9, 11]. Specific inhibition of CYP1A has been focused on the development of potential cancer preventative and therapeutic agents [6, 9, 12].

**Figure 1:**
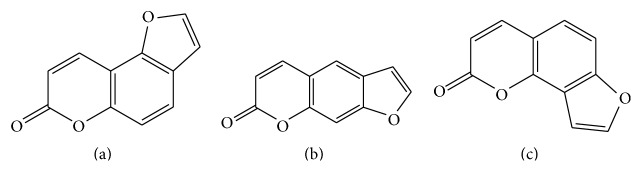
Chemical structures of bakuchicin (a), psoralen (b), and isopsoralen (c).

Bakuchicin is a furanocoumarin isolated from the seeds of* Psoralea corylifolia* which have been used in oriental medicine [13] (Figure 1). It has several biological effects including antivasorelaxant effect mediated by nitric oxide-cyclic GMP (NO-cGMP) signaling in rat aortic rings [14], antibacterial activity against a number of bacteria [15], and antitumor effect induced by strong DNA polymerase inhibition [16]. Although pharmacological effects of bakuchicin have previously been studied and selective inhibitory effects of its structurally similar analogs, psoralen and isopsoralen, have been investigated, the effects of its CYP activities have not been studied until now (Figure 1) [17]. Here, we investigated the selective inhibitory effect of bakuchicin on CYP1A in human liver microsomes (HLMs) and demonstrated its competitive inhibition.

## 2. Materials and Methods

### 2.1. Materials

Bakuchicin (chemical purities > 99.8%) was isolated from* P. corylifolia* (Figure 1) [13]. Pooled HLMs (BD UltraPool*™* HLM 150®) and human recombinant* cDNA-expressed* CYP1A1 and 1A2 were obtained from Corning Gentest (Woburn, MA). Phenacetin, coumarin, bupropion, paclitaxel, omeprazole, diclofenac, dextromethorphan, chlorzoxazone, midazolam, glucose 6-phosphate, *β*-nicotinamide adenine dinucleotide phosphate (*β*-NADPH), and glucose 6-phosphate dehydrogenase were purchased from Sigma-Aldrich (St. Louis, MO). All other chemicals were of analytical grade and were used as received.

### 2.2. Selective Inhibition of Bakuchicin on CYP1A

The inhibitory effects of bakuchicin on the metabolism of 8 different CYP-specific substrates are listed in Table 1 [18]. All incubations were performed in duplicate, and data are presented as means. Briefly, each reaction was performed with 0.5 mg/mL pooled human liver microsomes in a final incubation volume of 0.1 mL. The incubation medium consisted of 0.1 M potassium phosphate buffer (pH 7.4) containing cocktail of probe substrates and a NADPH-generating system (NGS) of 0.1 M glucose 6-phosphate, 10 mg/mL *β*-NADPH, and 1.0 U/mL glucose-6-phosphate dehydrogenase. To investigate the IC_50_ of bakuchicin during CYP1A inhibition, bakuchicin (at final concentrations of 0–25 *μ*M) was incubated without preincubation. In addition, the mixture with bakuchicin was preincubated for 15 min without the probe substrate cocktail in the presence or absence of 1 mM *β*-NADPH. After the reaction was initiated by adding the NGS for 60 min, incubated samples were terminated by adding 100 *μ*L acetonitrile containing 0.1% formic acid and 2.5 *μ*L of internal standard solution (5 *μ*M reserpine) in methanol. After mixing and centrifuging at 13,000 ×g for 12 min, a 5 *μ*L aliquot was injected onto a C18 column for LC-MS/MS analysis.

### 2.3. Inhibition Mode of Bakuchicin on CYP1A Activity in HLMs

To characterize the inhibition mode on CYP1A by bakuchicin, 0.5 mg/mL of HLMs was incubated with bakuchicin at 0, 0.2, 0.4, and 0.8 *μ*M in 0.1 M potassium phosphate buffer (pH 7.4) for 60 min at 37°C. Those were terminated by adding 100 *μ*L acetonitrile containing 0.1% formic acid and 2.5 *μ*L of internal standard solution (5 *μ*M reserpine) in methanol. After mixing and centrifuging at 13,000 ×g for 12 min, a 5 *μ*L aliquot was injected onto a C18 column for LC-MS/MS analysis. Phenacetin was used as a probe substrate at 20, 40, or 80 *μ*M. The inhibition pattern was investigated by Michaelis-Menten and its secondary plotting.

### 2.4. Inactivation Effects of Bakuchicin on Human Recombinant* cDNA-Expressed* CYP1A1 and CYP1A2

To confirm the selective inhibition of CYP1A by bakuchicin, 10 pmol of human recombinant* cDNA-expressed* CYP1A1 and CYP1A2 was incubated with bakuchicin at 0.1–50 *μ*M and NGS for 60 min at 37°C after the addition of 80 *μ*M phenacetin as a selective CYP1A substrate. Those were terminated by adding 100 *μ*L acetonitrile containing 0.1% formic acid and 2.5 *μ*L of internal standard solution (5 *μ*M reserpine) in methanol. After mixing and centrifuging at 13,000 ×g for 12 min, a 5 *μ*L aliquot was injected onto a C18 column for LC-MS/MS analysis. CYP1A1 and CYP1A2 activities were determined by phenacetin* O*-deethylation. The inhibition patterns of bakuchicin on CYP1A in human recombinant* cDNA-expressed* CYP1A1 and CYP1A2 were confirmed by Michaelis-Menten and secondary plot.

### 2.5. LC-MS/MS Analysis

All measurements were performed using LC-MS/MS in multiple reaction monitoring mode. LC-MS/MS was performed using an Accela*™* LC system coupled to a TSQ Vantage triple quadrupole mass spectrometer (Thermo Fisher Scientific Inc., USA) equipped with a HESI-II Spray source. Electrospray ionization was performed in positive mode at a spray voltage of 3,500 V (except for the detection of 6-hydroxy chlorzoxazone). Nitrogen was used as a sheath and auxiliary gas at optimum values of 45 and 20 (arbitrary units), respectively. Vaporizer and capillary temperatures were 150 and 300°C, respectively. For LC analysis an Inertsil® ODS-2 (3 *μ*m, 2.1 × 150 mm) column (GL Sciences Inc., Torrance, CA) was used. The mobile phase consisted of acetonitrile (mobile phase A) and water (mobile phase B), both of which contained 0.1% formic acid, at a flow rate of 0.22 mL/min at 30°C.

### 2.6. Data Analysis

All incubations were performed in duplicate, and data are presented as means. IC_50_ values (the concentration of inhibitor causing a 50% inhibition in enzyme activity) were obtained using percent activity versus log[*I*] concentration plots. Kinetic parameters were estimated by curve fitting using SigmaPlot (version 12.0, Systat Software, Inc.).

## 3. Results

### 3.1. Inhibitory Effects of Bakuchicin HLMs

To investigate the inhibitory effects of bakuchicin on the activity of 9 CYP isoforms, we conducted CYP inhibition assays using a cocktail of probe substrates coupled with an LC-MS/MS system. The IC_50_ values of bakuchicin on the 8 CYP activities were determined with or without microsomal preincubation for 15 min at 37°C to predict the inhibitory mechanism of bakuchicin. CYP2B and CYP2D6 were inhibited, but CYP1A was most strongly inhibited in HLMs compared to other CYPs at the IC_50_ value of 0.43 *μ*M (Table 1). Therefore, bakuchicin selectively inhibited CYP1A-catalyzed phenacetin* O*-deethylation in HLMs.

### 3.2. Competitive Inhibition of CYP1A by Bakuchicin in HLMs

**Figure 2:**
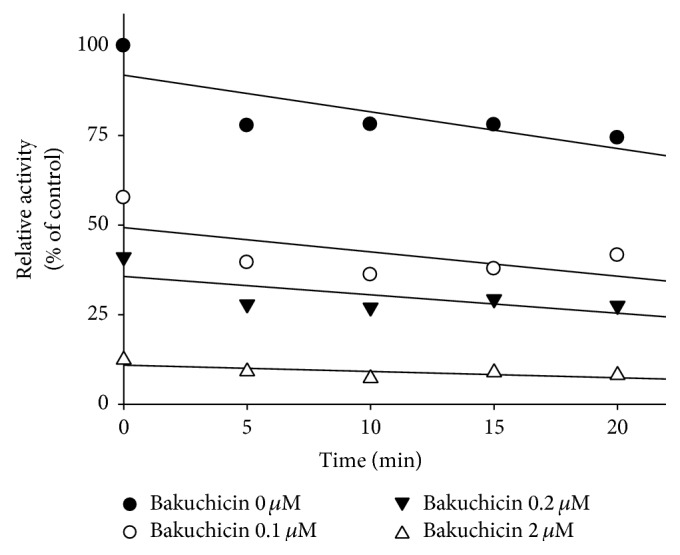
Time courses of CYP1A2-catalyzed phenacetin* O*-deethylation by bakuchicin in human liver microsomes (HLMs). HLMs were preincubated with bakuchicin at 0 (●), 0.1 (○), 0.2 (▼), and 2 (△) *μ*M of *β*-NADPH for 0 to 20 min. The results shown are the means of duplicate experiments.

In Table 1, the IC_50_ of bakuchicin on CYP1A-catalyzed phenacetin* O*-deethylation activity was determined with or without preincubation for 15 min at 37°C to investigate the inhibitory mechanism. When there was no preincubation, the IC_50_ value of bakuchicin on CYP1A was 0.43 *μ*M, whereas the IC_50_ value was 0.22 *μ*M after preincubation either with or without *β*-NADPH in pooled HLMs (Table 1). No significant change of IC_50_ after preincubation with or without *β*-NADPH indicates that bakuchicin is a reversible inhibitor of CYP1A-catalyzed phenacetin* O*-deethylation. In addition, to investigate the mechanism responsible for inhibition of CYP1A-catalyzed phenacetin* O*-deethylation by bakuchicin, CYP1A inhibitory activity was determined depending on preincubation time for 0–20 min at 37°C in HLMs. Bakuchicin strongly and dose-dependently inhibited CYP1A-catalyzed phenacetin* O*-deethylation, but not time-dependent inhibition in HLMs (Figure 2).

**Figure 3:**
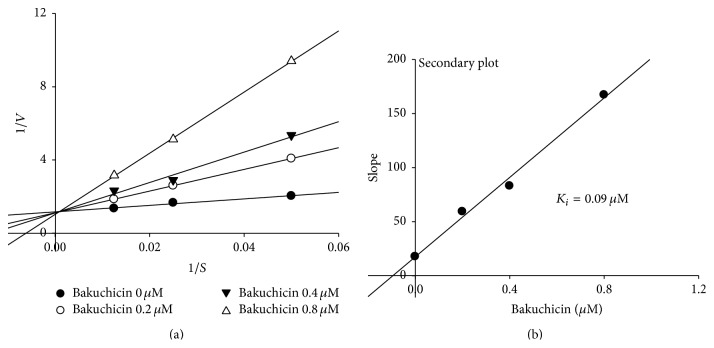
Lineweaver-Burk plot in pooled liver microsome obtained from a kinetic study of CYP1A-catalyzed phenacetin* O*-deethylation following 60 min of incubation with bakuchicin at 0 (●), 0.2 (○), 0.4 (▼), and 0.8 (△) *μ*M and phenacetin at 20, 40, and 80 *μ*M, respectively.

The mechanism of CYP1A inhibition of bakuchicin was determined using a Lineweaver-Burk plot (Figure 3). The kinetics were obtained by measuring CYP1A-catalyzed phenacetin* O*-deethylation in the presence of bakuchicin at 0, 0.2, 0.4, or 0.8 *μ*M (Figure 3(a)). Bakuchicin was found to be a reversible competitive inhibitor and the *K*_*i*_ value (0.09 *μ*M) was calculated from a secondary plot for CYP1A (Figure 3(b)).

### 3.3. Selective Inhibition of Bakuchicin on CYP1A1

**Figure 4:**
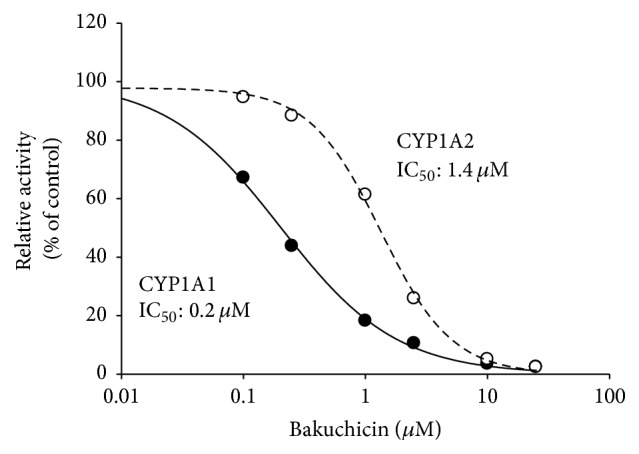
Inhibitory effects of bakuchicin on the phenacetin* O*-deethylation activities of human recombinant* cDNA-expressed* CYP1A1 and CYP1A2. The results shown are the means of duplicate experiments.

To investigate the selectivity of inhibitory effects of bakuchicin on CYP1A1 and CYP1A2, bakuchicin was incubated with human recombinant* cDNA-expressed* CYP1A1 and CYP1A2, respectively (Figure 4). Bakuchicin decreased CYP1A1-catalyzed phenacetin* O*-deethylase activity with an IC_50_ value of 0.2 *μ*M, whereas the IC_50_ was 1.4 *μ*M for CYP1A2-catalyzed phenacetin* O*-deethylase activity which was 7-fold higher than the value of CYP1A1 (Figure 4).

**Figure 5:**
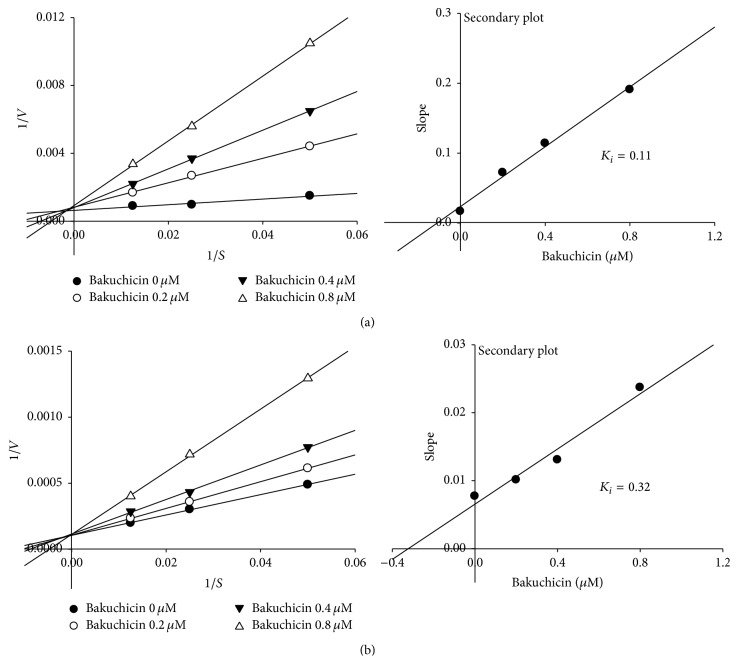
Lineweaver-Burk plot obtained from a kinetic study of CYP1A-catalyzed phenacetin* O*-deethylation following 60 min of incubation with bakuchicin at 0, 0.2, 0.4, and 0.8 *μ*M and phenacetin at 20 (●), 40 (○), and 80 (▼) *μ*M in human recombinant* cDNA-expressed* CYP1A1 (a) and CYP1A2 (b).

The inhibition modes of bakuchicin on CYP1A1 and CYP1A2 were confirmed by kinetic studies in human recombinant* cDNA-expressed* CYP1A1 and CYP1A2 (Figure 5). Lineweaver-Burk plots were obtained by a kinetic study of human recombinant* cDNA-expressed* CYP1A1 and CYP1A2-catalyzed phenacetin* O*-deethylation in the concentration of bakuchicin at 0, 0.2, 0.4, or 0.8 *μ*M, respectively (Figure 5(a)). The pattern showed that bakuchicin is a competitive inhibitor, and its *K*_*i*_ value on CYP1A1 calculated from a secondary plot was found to be 0.11 *μ*M, whereas its *K*_*i*_ value on CYP1A2 was 3-fold of that at 0.32 *μ*M (Figure 5(b)). This showed that bakuchicin inhibited CYP1A1 more selectively than CYP1A2 in HLMs.

## 4. Discussion

The CYP1A subfamily is well known to be involved in the bioactivation and detoxification of carcinogens [19]. CYP1A1 also plays an important role in the biotransformation of melatonin, arachidonic acid, eicosapentaenoic acid, dietary substrates, and environmental carcinogens [20–22]. CYP1A2 related to arylamine oxidation is important for demethylation of methylxanthines such as caffeine and theophylline,* O*-demethylation of naproxen, hydroxylation of tacrine, ropivacaine,* R*-warfarin and caffeine, dealkylation of phenacetin, and demethylation of tricyclic antidepressants [10, 23, 24]. Moreover, CYP1A2 is involved in the metabolism of medicines about 20% of clinically metabolized drugs such as those used in long-term treatment of schizophrenia and depression [25–27]. Because CYP1A is widely involved in drug metabolism, selective inhibition of CYP1A1 and CYP1A2 is important to prevent CYP-related drug-drug interactions with substrates that mostly undergo CYP1A-mediated metabolism.

In addition, CYP1A1 catalyzes the metabolic activation of carcinogenic PAHs to reactive electrophiles in rodents [28]. For examples, benzo[a]pyrene, dibenzanthracene, and benzo[h]fluoranthene are activated by CYP1A1 to form epoxide intermediates, which are further activated to form diol epoxides [29]. The metabolic activation of heterocyclic amines is also metabolized by CYP1A1 [10]. The hydroxylation at position N2 of 2-amino-1-methyl-6-phenylimidazo[4,5-b]pyridine (PhIP) by CYP1A1 initiates PhIP-DNA adduct formation, which induces carcinogenesis [11]. Therefore, the inhibition of CYP1A1 activity in order to inhibit the formation of CYP1A1-mediated carcinogenic reactive intermediates has been the focus on novel strategies in chemoprevention.

The inhibitory effect of bakuchicin on CYP1A was predicted from inhibitory effect of structural analogs, psoralen, and isopsoralen [17]. The psoralen and isopsoralen were identified as reversible and time-dependent inhibitor in human and rat liver microsomes. In the present study, we investigated selective inhibitory effects of bakuchicin on CYP1A in pooled HLMs. This was confirmed by selective inhibition of CYP1A-catalyzed phenacetin* O*-deethylation in HLMs, and human recombinant* cDNA-expressed* CYP1A1 and CYP1A2. In HLMs, bakuchicin showed the pattern of reversible competitive inhibitor with the *K*_*i*_ value of 0.09 *μ*M for CYP1A. In addition, the Lineweaver-Burk plot indicated the inhibition mechanism of bakuchicin to be competitive inhibition with *K*_*i*_ values of 0.11 *μ*M and 0.32 *μ*M in human recombinant* cDNA-expressed* CYP1A1 and CYP1A2, respectively. Understanding the basic mechanism of herb-drug interactions is useful not only in preventing drug toxicity, but also in developing novel cancer prevention therapies. This study showed that the metabolism of drugs that are CYP1A substrates, especially at high doses or when there is accumulation of the herb, may be important in metabolism-based herb-drug interactions [30, 31].

*P. corylifolia* is an important medicinal plant widely used in traditional medicine in China, India, and the United States as a cardiotonic, vasodilator, pigmentor, antitumor, antibacterial, cytotoxin, or anthelmintic [28]. Bakuchicin (1.51 g) was extracted from* P. corylifolia* fruit powder (300 g) by ethanol extraction [32]. The concentration of bakuchicin* in vivo* can depend on variability in the environment, the preparation method, or the patient. Therefore, it is important to identify the modulation effect of bakuchicin on CYP activities in HLMs. In conclusion, we were the first to investigate potential herb-drug interactions associated with bakuchicin by evaluating its inhibitory effects on CYP1A in HLMs. Our study showed that bakuchicin was a strong and selective competitive inhibitor of CYP1A1 and CYP1A2 in HLMs.

## Figures and Tables

**Table 1:** Inhibition of 9 CYP-catalyzed activities by bakuchicin in pooled human liver microsomes.

Substrate reaction probes	CYP450 isoforms	Substrate con. (*μ*M)	IC_50_ (*μ*M)		
			Without preincubation	With preincubation	
				Without NADPH	with NADPH
Phenacetin *O*-deethylation	CYP1A2	80	0.43	0.22	0.22
Bupropion hydroxylation	CYP2B6	50	14.2	16.4	11.5
Paclitaxel hydroxylation	CYP2C8	10	>100	>100	>100
Diclofenac 4′-hydroxylation	CYP2C9	10	>100	>100	>100
Omeprazole 5-hydroxylation	CYP2C19	20	>100	>100	>100
Dextromethorphan *O*-deethylation	CYP2D6	5	18.2	21.7	15.1
Chlorzoxazone 6-hydroxylation	CYP2E1	50	>100	>100	>100
Midazolam 1′-hydroxylation	CYP3A4	2.5	>100	>100	>100

To determine the inhibitory effects of bakuchicin on the activities of 8 CYPs, HLMs (0.05 mg) were preincubated with *β*-NADPH for 15 min and then with bakuchicin at 0 to 25 *μ*M for 60 min at 37°C. The data shown are the means of duplicates.
